# When delivery matters: Diffuse coronary vasospasm with continuous, but not bolus, 5-FU infusion

**DOI:** 10.21542/gcsp.2025.61

**Published:** 2025-12-31

**Authors:** Muhammad Salman Sabri, Zinya Talukder, Angad Bedi, Sung-Hae Cho

**Affiliations:** 1 Department of Internal Medicine, Jefferson Abington Hospital, Abington, PA, USA; 2 Department of Cardiology, Thomas Jefferson University Hospital, PA, USA

## Abstract

5-Fluorouracil (5-FU) is a fluorinated pyrimidine chemotherapy analogue used to treat various gastrointestinal solid malignancy. It is known to be associated with cardiotoxicity. Here, we present a case of 58-year-old male with mucinous adenocarcinoma of the sigmoid colon who experienced global coronary vasospasm in setting of continuous 5-FU infusion refractory to prophylactic antianginal treatment. However, he was asymptotic when given bolus of 5-FU. Our case highlights the difference in toxicity between continuous and bolus 5-FU therapy and emphasizes the need for further research in the pathophysiology and treatment of cardiotoxicity related to 5-FU therapy.

## Introduction

5-fluorouracil (5-FU) is a fluorinated pyrimidine analogue used for gastrointestinal cancer such as colorectal, esophageal, stomach and pancreatic cancers. It inhibits thymidylate synthase resulting in depletion of intracellular thymidylate needed for deoxyribonucleic acid (DNA) synthesis. There are various cases in literature documenting diffuse coronary vasospasm linked to the administration of 5-FU continuous infusion^1^. Case reports have also described incidence of no coronary vasospasm with bolus 5-FU^2,3^. We present a case of global coronary vasospasm in setting of 5-FU chemotherapy refractory to prophylactic treatment with calcium channel blocker and nitrate.  We discuss the difference in toxicity and tumor response between continuous and bolus 5-FU therapy. Our discussion emphasizes the need for further research in the pathophysiology, risk factors, diagnostic approaches, and treatment of cardiotoxicity related to 5-FU therapy. 

### Case report

**Figure 1.**
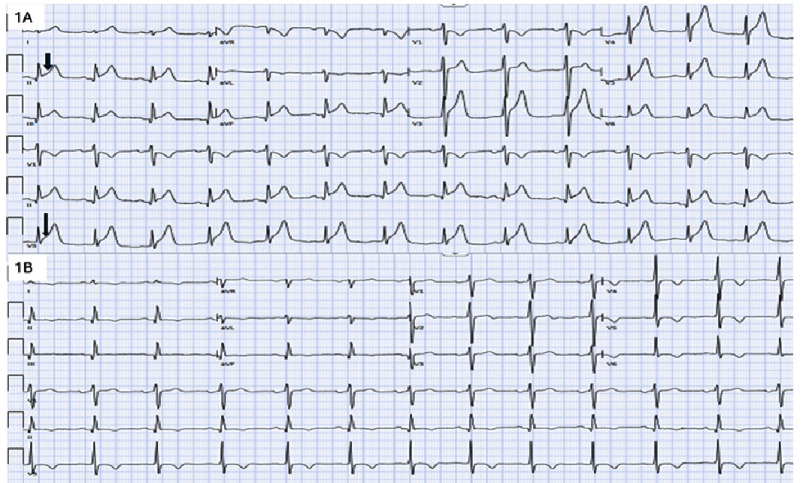
Electrocardiogram (1A) demonstrate ST-segment elevations in inferior, anterior and lateral leads. There is also ST-segment depression in lead AVR. Repeat electrocardiogram (1B) demonstrates T wave inversions in anterolateral and inferior leads. There are also Q waves in inferior leads.

A 58-year-old male with hypertension, hyperlipidemia, obstructive sleep apnea, and mucinous adenocarcinoma of the sigmoid colon (pT3pN2b cM0, Stage IIIC) status post R0 sigmoid colectomy, presented with chest pain during outpatient 5-fluorouracil (5-FU) chemotherapy. Given his high-risk pathological features, he was initiated on adjuvant FOLFOX (Folinic Acid, Fluorouracil, and Oxaliplatin). Approximately 30 h into a continuous 5-FU infusion, he developed anginal chest pain. Initial electrocardiogram (ECG) revealed ST-segment elevations in the anterior leads, lateral leads and inferior leads [Figure 1A].

His vital signs were notable for a blood pressure of 140 mmHg systolic, heart rate of 70 beats per minute, and oxygen saturation of 98% on room air. Physical examination revealed normal heart sounds without murmurs or gallops, clear lung fields, and no peripheral edema.

**Figure 2.**
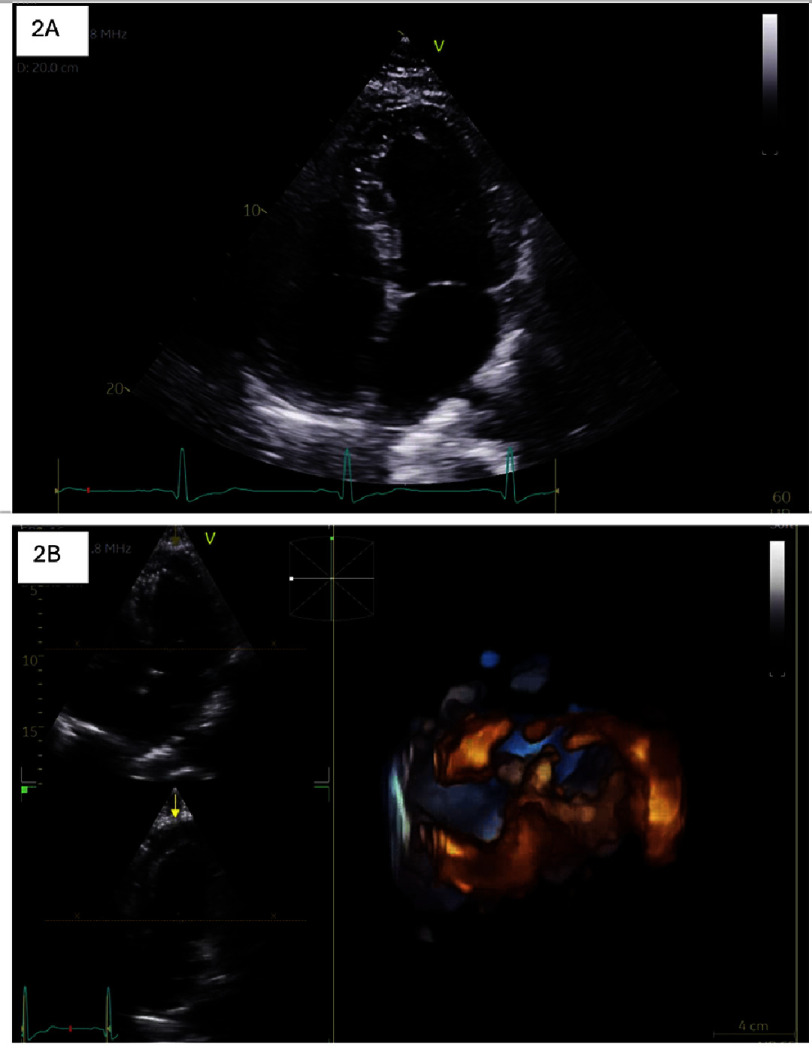
Transthoracic echocardiogram demonstrated normal left and right ventricular size and function. The left atrium was noted to be mildly to moderately dilated.

Laboratory evaluation showed a troponin peak of 17 ng/mL, which down-trended to 11 ng/mL two hours later. Transthoracic echocardiography (TTE) [Figure 2] showed a normal-sized left ventricle with concentric remodeling and low-normal systolic function, with an ejection fraction of 54% by biplane Simpson method. Global longitudinal strain (GLS) was normal at −20%. The right ventricle was normal in size and function. There was no evidence of significant valvular stenosis or regurgitation. Coronary angiography demonstrated no obstructive coronary disease, and the presentation was consistent with 5-FU–induced coronary vasospasm.

After multidisciplinary discussion with oncology and cardiology, decision was made to rechallenge the patient with 5-FU with pre-treatment of calcium channel blockers and nitrates. Despite premedication with both dihydropyridine and non-dihydropyridine calcium channel blockers and nitrates, chest pain and diffuse ST elevations recurred when he was rechallenged with continuous 5-FU infusion. A decision then was made to re-try the 5-FU as a bolus, rather than a continuous infusion, along with pretreatment with vasodilators. The patient was subsequently given a bolus of 5-FU, administered as a 12-minute infusion, following pretreatment with oral diltiazem 60 mg, nifedipine 10 mg, and isosorbide mononitrate 120 mg. He was closely observed during the 12-minute infusion and for 15 min after the infusion.

**Figure 3.**
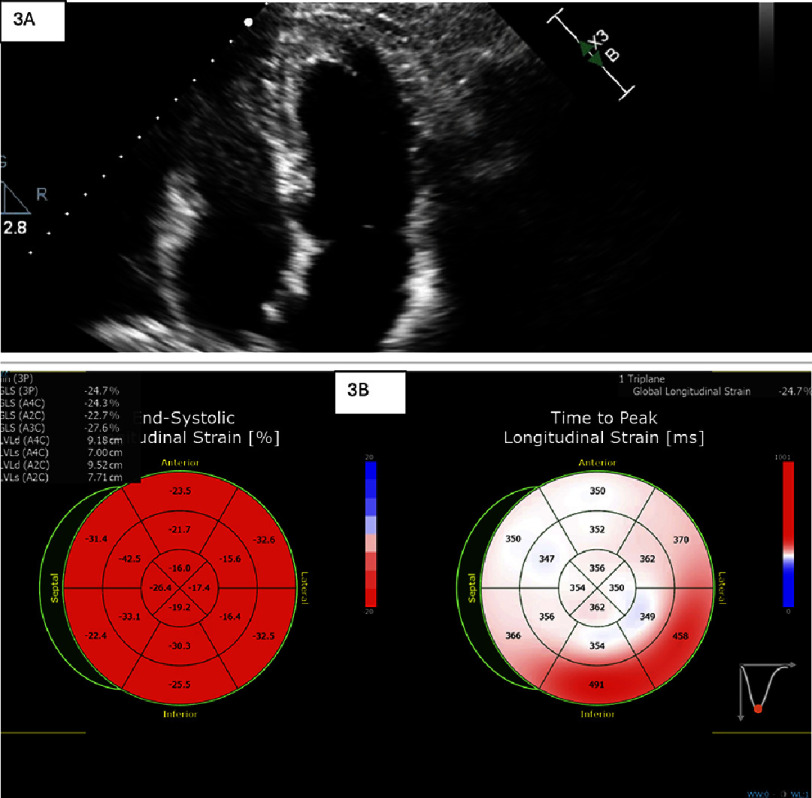
Transthoracic echocardiogram demonstrated normal left ventricular systolic function and chamber dimensions (Figure 3A). Global longitudinal strain was also within normal limits, as shown in Figure 3B.

**Figure 4.**
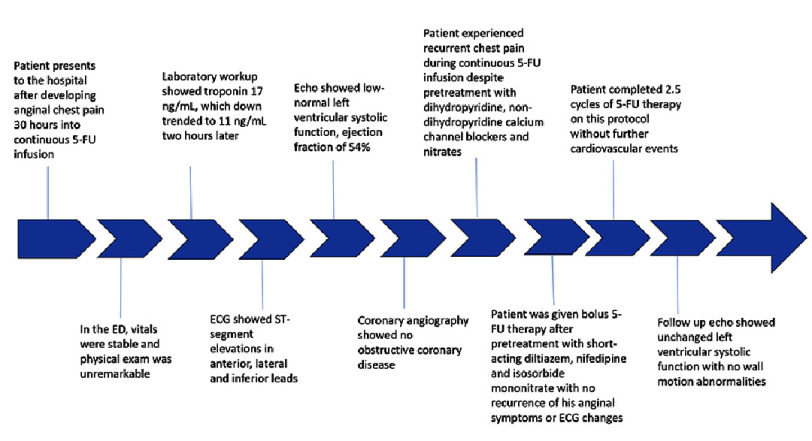
Visual summary of the patient’s clinical course.

He tolerated the bolus 5-FU without recurrence of chest pain or ECG changes, which suggested that there may be reduced vasospasm with bolus administration compared to continuous infusion. He successfully completed 6 months of 5-FU bolus as part of FLOX (2.5 cycles) of adjuvant chemotherapy under this protocol without further cardiovascular events. Each cycle comprises 3 days of infusion and the next 11 days are rest. A follow-up TTE [Figure 3] showed that left ventricular systolic function remained normal, with an LVEF of 58% and GLS of −24.7%, and no regional wall motion abnormalities. The patient continues to be followed by cardio-oncology and medical oncology and remains asymptomatic from a cardiovascular standpoint. He underwent surveillance CT chest abdomen and pelvis without evidence of recurrent disease. In addition, the patient’s most recent revealed a single three mm tubular adenoma in the proximal rectum. The patient is scheduled to have surveillance colonoscopy every 3 years. See Figure 4 for a timeline of this case.

## Discussion

5-FU is one of the most common chemotherapeutic drugs associated with cardiotoxicity after anthracyclines and trastuzumab. The incidence of cardiotoxicity with 5-FU is 1.6–8.5%. In terms of the various types of cardiotoxicities associated with 5-FU, 45% are associated with angina, 22% myocardial infarction, 22% arrhythmias, 5% acute pulmonary edema, 2% heart failure, and 1.4% cardiac arrest and pericarditis^4^. 5-FU cardiotoxicity tends to occur most commonly during the first cycle of administration and the median time to onset of symptoms is 12 h. However, cardiotoxicity can occur anytime during the infusion or even up to 1–2 days after infusion^5^. Our patient developed anginal chest pain 30 h into a continuous 5-FU infusion.

### Mechanism of 5-FU cardiotoxicity

Although the precise mechanism of 5-FU cardiotoxicity remains unclear, proposed mechanisms include coronary artery vasospasm, direct toxicity to the myocardium, endothelial dysfunction and hypercoagulable state leading to thrombosis. Coronary vasospasm leading to an acute ischemic event is a well-recognized cardiac side effect of 5-FU. Patients typically present with signs and symptoms of acute coronary syndrome with elevations and elevated cardiac biomarkers. Coronary angiography should be performed, but they are typically normal without evidence of thrombosis^6^.

### Risk factors

Risk factors for cardiotoxicity in patients taking 5-FU is not precisely understood either. Several studies suggest that age greater than 55 years, pre-existing renal and cardiac disease are potential risk factors. However, other studies have shown that most patients experiencing cardiotoxicity do not necessarily have pre-existing cardiac disease^5^. Our patient was greater than 55 years of age, with hypertension and hyperlipidemia as additional risk factors. Further research needs to be performed to determine whether pre-existing renal and cardiac disease, along with other cardiac risk factors such as diabetes, hypertension, hyperlipidemia, and smoking, increase a patient’s risk for cardiotoxicity.

### Differences between administration methods

5-FU is one of a minority of clinical drugs whose spectrum of toxicity differs based on doses and schedules. Different methods of administration have been shown to produce significantly different toxicity, particularly when bolus schedules are compared to continuous infusion schedules. Bolus dosing has been associated with myelosuppression as its major toxicity with mucositis and diarrhea as its minor toxicities; low dose continuous infusion has been associated with palmar-plantar dysesthesia and high dose continuous infusion has been associated with mucositis^7^.

A meta-analysis performed on the efficacy of intravenous continuous infusion (CI) of 5-FU compared to bolus administration in patients with advanced colorectal cancer, showed that tumor response rate was significantly higher in patients assigned to 5-FU CI than in patients assigned to 5-FU bolus, 22% versus 14%, respectively. In addition, overall survival was significantly higher in patients assigned to 5-FU CI group. Hematologic toxicity was more common in the bolus group whereas hand-foot syndrome occurred more frequently in the CI group^8^.

Although there are no randomized data, few case reports have suggested that bolus administration of 5-FU may have less cardiotoxicity than continuous administration of 5-FU^1–3^. Our patient with mucinous adenocarcinoma of sigmoid colon had coronary vasospasm with diffuse ST elevations on continuous infusion of 5-FU but was able to tolerate the bolus dose of 5-FU without recurrence of symptoms.

The difference in toxicity may be attributable to pharmacokinetic variations between the two methods: 5-FU has a short plasma half-life of 15 to 20 min and is rapidly cleared from circulation. Bolus dosing results in a transient exposure to the drug, whereas continuous infusion maintains prolonged systemic levels, potentially leading to greater accumulation and higher cardiotoxic risk.

### Management strategies

Treatment of 5-FU induced coronary vasospasm involves immediate discontinuation of chemotherapy then treating with antianginal agents such as calcium channel blockers or nitrates^5^. It is important to determine if the patient’s symptoms can be reasonably attributed to 5-FU. In addition, alternative underlying cardiac pathological processes must be ruled out.

Ancillary testing such as ECG, echocardiography, coronary angiography, and laboratory testing such as cardiac enzymes and brain natriuretic peptide are commonly used to aid clinical judgement. The presence of significant coronary stenosis should not exclude the possibility of superimposed 5-FU related cardiotoxicity.

In our case, invasive pharmacologic provocation for vasospasm at coronary angiography could have been considered. For patients who develop vasospasm from 5-FU, but for whom 5-FU remains the preferred chemotherapy agent, pretreatment with vasodilatory medications such a calcium channel blockers and nitrates, prior to continuous infusion may reduce the risk of recurrent cardiotoxicity^1^.

Our case is distinctively unique as our patient continued to experience angina despite pretreatment with dihydropyridine and non-dihydropyridine CCBs and nitrates. However, he did not experience any anginal symptoms when given 5-FU bolus therapy, suggesting that a bolus regimen may offer a safer alternative to continuous infusion in select cases.

The failure of prophylactic vasodilators during continuous 5-FU infusion is likely multifactorial. While vasospasm is a well-recognized mechanism of 5-FU cardiotoxicity, it is not the only one. 5-FU also induces endothelial oxidative stress, leading to endothelial dysfunction and cell death, direct myocardial injury, and microvascular dysfunction. Therefore, although vasodilators may help prevent epicardial vasospasm, they have limited effect on these additional mechanisms of injury and thus do not eliminate the overall risk of 5-FU–related cardiotoxicity^9,10^.

## Conclusion

This case of a 58-year-old male with mucinous adenocarcinoma of the sigmoid colon who experienced global coronary vasospasm in setting of continuous 5-FU infusion refractory to prophylactic antianginal treatment. However, asymptotic when given bolus of 5-FU highlights the potential for severe cardiotoxicity, particularly coronary vasospasm, associated with continuous 5-FU infusion. In addition, it supports the findings of several other case reports that patients with prior vasospastic reactions, pretreatment with vasodilators and switching to a bolus regimen may offer a safer and more tolerable alternative. However, further randomized data is needed to definitively establish superiority of bolus administration of 5-FU from continuous infusion of 5-FU.

## Limitations

Some limitations of our case includes although our patient reported no anginal symptoms and no ECG changes were observed during bolus administration of 5-FU no coronary angiography was obtained to determine vasospasm during bolus administration.

## Disclosures

The authors have nothing to disclose.

## Funding

The authors received no funding to disclose.

## References

[1] Yildirim M, Parlak C, Sezer C, Eryilmaz R, Kaya C, Yildiz M (2011). Coronary vasospasm secondary to 5-Fluorouracil and its management: case report. Eurasian J Med.

[2] Shaib W, Lee V, Saif MW (2009). Bolus 5-fluorouracil as an alternative modality to infusion 5-fluorouracil in a patient with rectal cancer and capecitabine-induced cardiotoxicity. Vivo.

[3] Saif MW, Garcon MC, Rodriguez G, Rodriguez T (2013). Bolus 5-fluorouracil as an alternative in patients with cardiotoxicity associated with infusion 5-fluorouracil and capecitabine: a case series. In Vivo.

[4] Saif MW, Shah MM, Shah AR (2009). Fluoropyrimidine-associated cardiotoxicity: revisited. Expert Opin Drug Saf.

[5] Sara JD, Kaur J, Khodadadi R, Rehman M, Lobo R, Chakrabarti S, Herrmann J, Lerman A, Grothey A (2018). 5-fluorouracil and cardiotoxicity: a review. Ther Adv Med Oncol.

[6] Yuan C, Parekh H, Allegra C (2019). 5-FU induced cardiotoxicity: case series and review of the literature. Cardio-Oncology.

[7] Macdonald JS (1999). Toxicity of 5-fluorouracil. Oncology (Williston Park).

[8] (1998). Efficacy of intravenous continuous infusion of fluorouracil compared with bolus administration in advanced colorectal cancer. Meta-analysis Group In Cancer. JCO.

[9] Fabin N, Bergami M, Cenko E, Bugiardini R, Manfrini O (2022). The role of vasospasm and microcirculatory dysfunction in fluoropyrimidine-induced ischemic heart disease. J Clin Med.

[10] Focaccetti C, Bruno A, Magnani E, Bartolini D, Principi E, Dallaglio K, Bucci EO, Finzi G, Sessa F, Noonan DM, Albini A (2015). Effects of 5-fluorouracil on morphology, cell cycle, proliferation, apoptosis, autophagy and ROS production in endothelial cells and cardiomyocytes. PLOS ONE.

